# New challenges in the study of the evolution of wild animals and their gut microbiome

**DOI:** 10.1002/ece3.8904

**Published:** 2022-05-07

**Authors:** Lifeng Zhu

**Affiliations:** ^1^ 12534 College of Life Sciences Nanjing Normal University Nanjing China

**Keywords:** behavior, environment, gut microbiome, host bias, strain level, the wild animal

## Abstract

In this viewpoint, by reviewing the recent findings on wild animals and their gut microbiomes, we found some potential new insights and challenges in the study of the evolution of wild animals and their gut microbiome. We suggested that wild animal gut microbiomes may come from microbiomes in the animals' living habitats along with animals' special behavior, and that the study of long‐term changes in gut microbiomes should consider both habitat and special behaviors. Also, host behavior would facilitate the gut microbiome transmission between individuals. We suggested that research should integrate the evolutionary history and physiological systems of wild animals to understand the evolution of animals and their gut microbiomes. Finally, we proposed the Noncultured‐Cultured‐Fermentation‐Model Animal pipeline to determine the function (diet digestion, physiology, and behavior) of these target strains in the wild animal gut.

## Graphical abstract

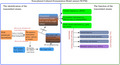

## 1 INTRODUCTION

Over the past two decades, there have been many studies on the evolutionary relationship between animals and their gut microbiomes (Ley et al., 2008; Park, 2018; Wei et al., 2019). Animal gut microbiomes play important roles in their hosts’ development and health (Ley, Lozupone, et al., 2008; Park, 2018). Diet, phylogeny, and genetics of the host are the main factors that influence the composition and function of the gut microbiome (Bonder et al., 2016; Goodrich et al., 2014; Grieneisen et al., 2021; Ley, Hamady, et al., 2008; Ley, Lozupone, et al., 2008; Park, 2018; Wang et al., 2021). Recently, an increasing amount of research has revealed that animal social behaviors (e.g., grooming, cuddling, mating, and other social contacts) also shape the gut microbiome community (Ezenwa et al., 2012; Li et al., 2021; Moeller et al., 2016; Nagpal & Cryan, 2021; Tung et al., 2015; Xia et al., 2022; Zhu et al., 2020). Environment (e.g., shared household or cohabitating) has a profound role in shaping animal gut microbiome (Gacesa et al., 2022; Rothschild et al., 2018; Song et al., 2013). Animals exhibit many kinds of behavior in addition to the behaviors necessary to survive and reproduce. What are the potential effects on their symbiotic microbiomes?

## 2 THE MICROBIOME TRANSMISSION BETWEEN WILD ANIMALS AND THEIR ENVIRONMENT ALONG WITH SEASONAL BEHAVIOR

**FIGURE 1:**
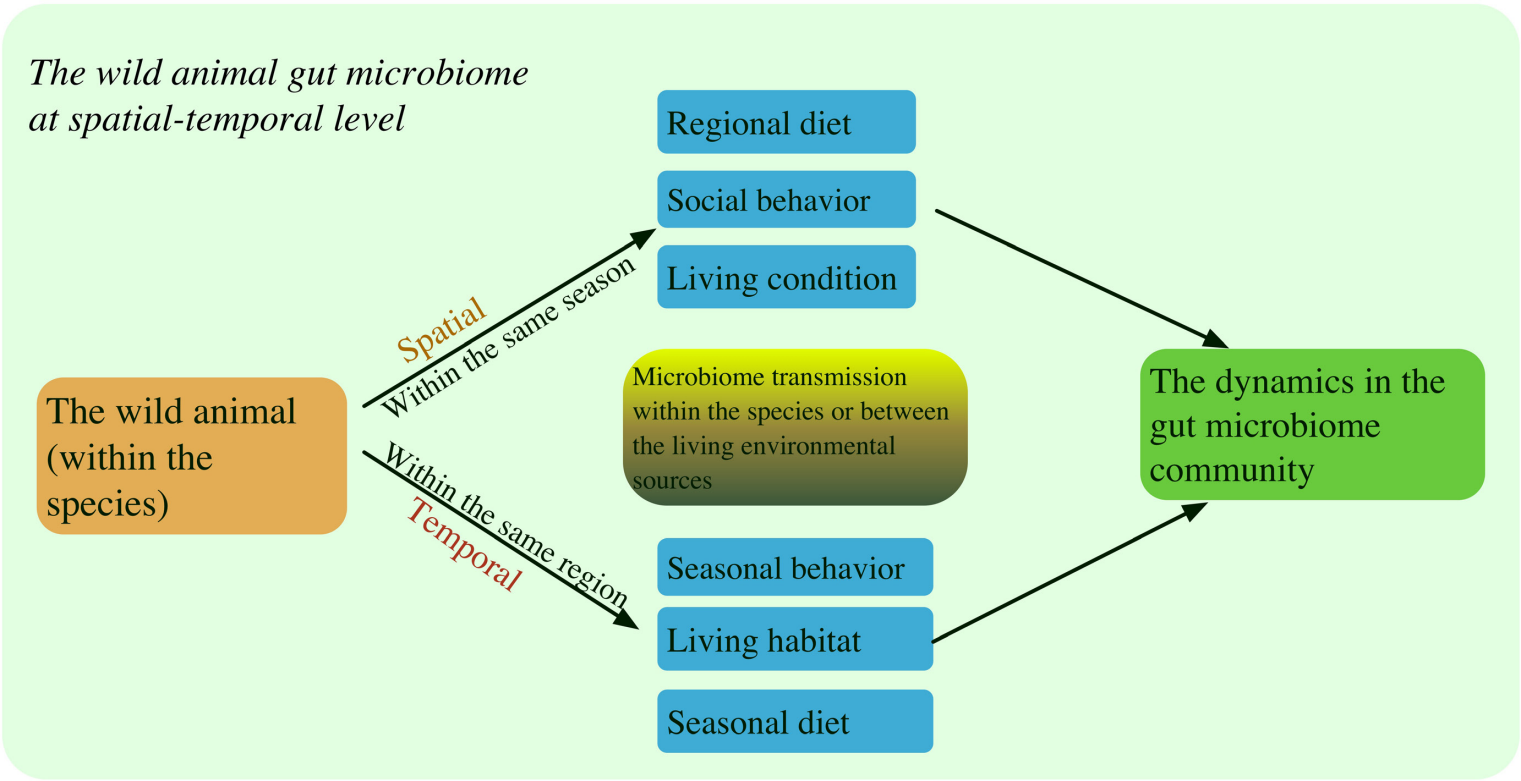
The wild animal gut microbiome at the spatial‐temporal level. Here, we display the potential effect on the gut microbiome community by host diet, behavior, and living condition

More and more studies have investigated the gut microbiome transmission between individuals during the course of their social behaviors (Ezenwa et al., 2012; Moeller et al., 2016; Tung et al., 2015). However, animal gut microbiota may also come from their living environment. The first question we must ask is: What proportion of animal gut microbiota comes from animals’ habitats? Some studies have found that the seasonal changes in the animal gut microbiome community are due to dietary changes in different seasons (Hicks et al., 2018; Reese et al., 2021; Wu et al., 2017). Following season‐specific fecal microbiota transplantation, mice transplanted with the microbiota from the giant panda in the shoot‐eating season grew faster and stored more fat (Huang et al., 2022). Animals may display seasonal behavior and make local adaptations to their diet and behavior to ensure their survival. Thus, the second question here is: What are the effects of seasonal behavior on the gut microbiome community, not just considering seasonal changes in diet? For example, amphibians are poikilothermic animals, and they are sensitive to changes in the natural environment. When the temperature rises in the spring, they can migrate to ponds or wetlands to breed (Figiel & Semlitsch, 1995; Freda, 1983; Licht, 1969). During the winter, they migrate to hibernation sites (e.g., caves) to maintain their body temperature. Their seasonal diets are similar (Figiel & Semlitsch, 1995; Freda, 1983; Licht, 1969). Thus, wild amphibians are a suitable model to quantify the effects of living habitats along with seasonal behavior on gut microbiomes. One study found that about 20% of the amphibian gut microbiome in spring may have come from water sources, while only about 5% came from water sources in autumn (Xu et al., 2020). This indicated that wild animal gut microbiomes may be significantly impacted by the microbiomes in the environment along with special seasonal behaviors (Xu et al., 2020). Research on long‐term changes in animal gut microbiomes have to consider both animals’ living environments and special behaviors (Figure 1).

**FIGURE 2:**
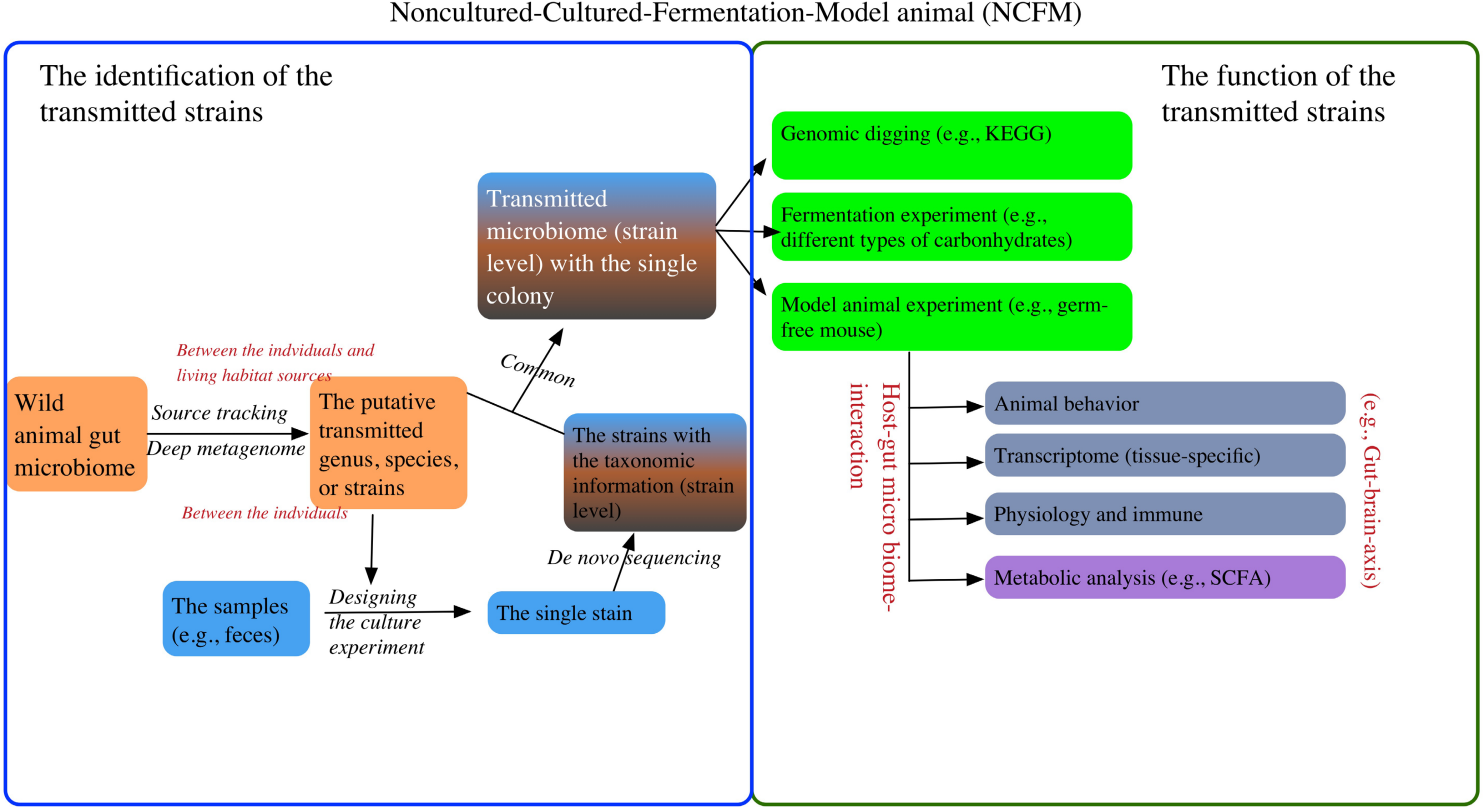
Future perspectives in the study of the evolution of wild animals and their gut microbiomes: Noncultured‐Cultured‐Fermentation‐Model Animal (NCFM). This research frame included the identification of the transmitted strains and the downstream analysis on the function of these transmitted strains. SCFA, Short‐chain fatty acids. KEGG, Kyoto Encyclopedia of Genes and Genomes

There are many animal species in the natural world. These species display a rich variety of behavior within and between the species in the ecosystem. Thus, when designing research plans to investigate the wild animal gut microbiome community and origin, researchers should consider both the potential microbiome transmission between the species and microbiome sources in their living environments (Figure 2). Beyond the vertical microbiome transmission (mother to offspring) (Colston, 2017; Wang et al., 2020), the special characters (e.g., behavior and physiological characters) of studied species may further shape their gut microbiome community under similar diets. The investigation and understanding of the wild animal gut microbiome community at the spatial‐temporal level should integrate the hosts’ diet, seasonal behavior, and living habitats (Figure 1).

## 3 CONFLICTING SYMBIOTIC SYSTEM—A CHALLENGE TO THE CURRENT CONSENSUS ON THE EFFECTS OF DIET ON THE STABILITY OF THE WILD ANIMAL GUT MICROBIOME

There are many examples that show the stability of the animal gut microbiome given the same or similar diet (Lozupone et al., 2012). Within species, diet is one of the most important factors leading to stable gut microbiome composition and function. However, we have found a challenge to this school of thought based on the long‐term monitoring of wild and captive giant panda gut microbiomes (Yao et al., 2019). Although they have similar diets (bamboos), the gut microbiome system in giant pandas is unstable and the gut microbiome community and its associated functions are highly variable and differ (Yao et al., 2019). Bamboo‐eating pandas (giant pandas and red pandas) shared some similar gut microbial features (e.g., a high proportion of Pseudomonas) (Huang et al., 2021; Yao et al., 2019). We speculate that difficult‐to‐digest bamboo coupled with pandas’ simple carnivorous digestive systems is the cause of this unstable microbiome community (Yao et al., 2019). Therefore, we reconsider the relationship between the wild animal gut microbiome community and their diet along with the specific character of the host evolutionary history (Yao et al., 2019, 2021). This finding in giant pandas provides us with an example of a conflicting symbiotic system and its trade‐off: the giant panda gut microbiome has been adapted to its bamboo diet and aids in digesting cellulose and detoxifying cyanide compounds; and the bamboo diet also is the disturbance leading to high variation in the giant panda gut microbiome community (Yao et al., 2019, 2021; Zhang et al., 2020). Moreover, we have revealed the host bias in the diet‐source microbiome in the cohabitating herbivores, and the herbivorous insect gut microbiome may be mainly from the diet‐source microbiome, rare in the deer; and this could be caused by the difference in the oxygen level in their intestines (Zhu et al., 2021). Thus, we have to re‐think the evolution of herbivore and plant defense. Beyond the enzymes from the host themselves, the simplest and superior way that harboring dietary plant symbiotic microbiome would be beneficial for the adaptation to host–plant interaction.

## 4 FUTURE PERSPECTIVES IN THE STUDY OF THE EVOLUTION OF WILD ANIMALS AND THEIR GUT MICROBIOMES: NCFM

According to the current findings on wild animal gut microbiomes, one of the main challenges in this line of research is determining the function of these transmitted microbiome strains (within the species or between the living environment and the species) (Figure 2). Most studies are based on high‐throughput sequencing methods (e.g., 16s rRNA MISEQ and metagenome) and only know the putative bacterial genus and the low‐quality assembled genomes. Thus, future lines of research may go back to cultured methods. First, we can use the current deep‐metagenomic and binning method to assemble high‐quality transmitted microbial strains. Second, at the same time, based on the putative transmission strains, we can use the cultured method to collect the strain from the samples, and further use de novo sequencing to determine their putative function (gene‐level). Third, we can transplant the target strain into the model animal (e.g., germ‐free mice) to reveal the putative effects on the host physiology and behavior, and we can discuss the interaction between host and gut microbiome (e.g., the gut–brain axis). Fourth, we can also use fermentation experiments (using different kinds of carbohydrates) on these target strains to understand their function in digestion.

Noncultured‐Cultured‐Fermentation‐Model Animal (NCFM) is not solely limited to research on transmitted microbiomes, but it is also suitable for studies on microbiomes in the animal gut. This method will allow us to determine the function (diet digestion, physiology, and behavior) of these target strains and host–gut microbiome interaction (e.g., gut–brain axis), which will help us to understand evolutionary adaptations in the animal and their gut microbiome.

## CONFLICT OF INTEREST

The authors declared no conflicts of interest relevant to this manuscript.

## AUTHOR CONTRIBUTION

**Lifeng Zhu:** Conceptualization (lead); Writing – original draft (lead).

## Data Availability

Not applicable. This manuscript didn't contain DNA sequences and other unpublished data.
